# Progress Towards Measles Elimination in Nigeria: 2012 – 2016

**Published:** 2018-08-02

**Authors:** Balcha Masresha, Fiona Braka, Nneka Ukachi Onwu, Joseph Oteri, Tesfaye Erbeto, Saliu Oladele, Kyandindi Sumaili, Abimbola Aman-Oloniyo, Regis Katsande, Sisay Gashu Tegegn, Amadou Fall

**Affiliations:** 1 World Health Organisation- Regional office for Africa. Brazzaville, Congo; 2 World Health organisation – Country office for Nigeria. Abuja, Nigeria; 3 National Primary Health Care Development Agency, Nigeria; 4 United Nations Children’s Fund (UNICEF) - Country Office for Nigeria. Abuja, Nigeria; 5 World Health Organisation- Inter-country support team for West Africa. Ouagadougou, Burkina Faso

**Keywords:** Measles Elimination, Progress, Nigeria

## Abstract

**Introduction:**

Nigeria has adopted the African Regional measles elimination targets and is implementing the recommended strategies. Nigeria provides routine measles vaccination for children aged 9 months. In addition, since 2006, Nigeria has been conducting nationwide measles supplemental Immunisation activities (SIAs) or mass vaccination campaigns every 2 years, and has established measles case-based surveillance.

**Methods:**

We reviewed routine and supplemental measles immunization coverage data, as well as measles case-based surveillance data from Nigeria for the years 2012 – 2016, in an attempt to determine the country’s progress towards these elimination targets.

**Results:**

The first dose measles vaccination coverage in Nigeria ranged from 42% and 54% between 2012 and 2015, according to the WHO UNICEF national coverage estimates. Nigeria achieved 84.5% coverage by survey following the 2015 nationwide measles supplemental immunisation activities (SIAs). During this period, the incidence of confirmed measles ranged from 25 - 300 confirmed cases per million population per year, with the Northern States having significantly higher incidence as compared to the Southern States. At the same time, the pattern of confirmed cases indicated a consistent shift in epidemiological susceptibility including older age children.

**Conclusions:**

In order to accelerate its progress towards the measles elimination targets, Nigeria should build population immunity on a sustainable basis by addressing systemic issues in order to scale up routine immunisation coverage, especially in the Northern half of the country; tailoring the target age for measles SIAs so as to sharply reduce measles incidence in age groups heavily affected by the disease; effectively mobilising resources and improving the quality of planning and coverage outcome of SIAs.

## Introduction

The 47 Member States of the African Region of the World Health Organization established a goal in 2011 to achieve measles elimination by 2020 with the following targets 1) ≥95% coverage with the first dose of measles-containing vaccine (MCV1) at national and district levels, 2) ≥95% supplemental immunization activity (SIA) coverage in every district, and 3) confirmed measles incidence of <1 per million population in all countries, and attaining the targets for the two principal surveillance performance monitoring indicators which are: ≥80% of districts with ≥1 suspected measles case with blood specimen reported per year and a non-measles febrile rash illness rate of ≥2 per 100,000 population^1^.

WHO recommends reaching all eligible children with two doses of measles vaccine in the routine immunisation service. It is recommended that, in high incidence settings, the two doses of measles vaccine are provided at 9 months of age and in the second year of life respectively^2^. Case based surveillance for measles was established in order to monitor the impact of the vaccination activities, and this was implemented using the infrastructure already available for polio surveillance. The suspected measles case definition is generalized maculo-papular rash and fever plus one of the following: cough, coryza (runny nose), or conjunctivitis. All suspected measles cases are investigated by the health facility and district levels, and specimens collected for serological confirmation, unless in the case of confirmed outbreaks, when confirmation of cases can be done by epidemiological linkage^3^.

The SIA strategy of periodic nationwide mass vaccination campaigns is introduced in order to ensure that children missed in routine immunisation services are given the opportunity to be vaccinated. SIAs are used to reach all children in the target age group regardless of their vaccination status and include special planning to reach subpopulations that do not access routine vaccination services. The strategy starts with a one-time catch-up campaign targeting children aged 9 months to 14 years, followed by periodic follow-up campaigns conducted every 2–4 years that target children who were born after the previous SIA. Generally, follow-up SIAs target children aged 9 months to 5 years, depending on the interval between the SIAs^4^.

With the implementation of these recommended strategies, the African Region of the WHO has achieved 85% reduction in estimated measles deaths by the end of 2015 as compared to mortality estimates in 2000^5^. However, the implementation of the strategies and the progress between 2013 – 2016 indicates that the African Region is not on track to meet the 2020 measles elimination targets^6^.

Nigeria is divided into 36 states and 1 Federal Capital Territory (FCT). The states are grouped into six geopolitical zones; South-South, South East, South West, North East, North West and North Central. The Northern zones comprise of 19 States and FCT, while there are 17 States in the Southern part of the country.

Nigeria introduced measles vaccination into the routine immunisation program in 1978 for children aged 9 months. DHS data from 2013 indicated that only 42% of children 12 – 23 months had received measles vaccine, and that there is a great disparity in the measles vaccination coverage levels among the various geopolitical zones, with the three Northern zones having measles vaccination coverage of 48.1%, 26.8% and 22.3% for North Central, North East and North West respectively. On the other hand, South East, South South and South West zones had coverage of 72.2%, 74% and 62.5% respectively^7^.

Nigeria started accelerated measles control activities in 2006 with the conduct of the first catch-up measles campaign in the northern States, followed by the southern part of the country. Owing to the low immunisation coverage, the high volume of the birth cohort and the fast accumulation of susceptible children, Nigeria has been conducting nationwide mass vaccination campaigns every 2 years.

In 2006, Nigeria established a system of measles case-based surveillance as per the WHO guidelines. Four subnational measles serological laboratories were established across the country to test all specimens from suspected cases of measles for measles IgM antibodies. Specimens with negative measles Igm results are tested for rubella IgM.

Nigeria has adopted the regional measles elimination targets as articulated in a National Measles Elimination Plan 2012-2016, and updated draft plan for 2017-2020^8^. Nigeria does not yet have a second dose of measles vaccine in its routine immunisation schedule (MCV2). The latest WHO position paper on measles vaccines recommends that all countries should include MCV2 in their national vaccination schedules regardless of the level of MCV1 coverage^9^. This report describes progress toward measles elimination in Nigeria during 2012–2016, since the adoption of the Regional measles elimination goal.

## Methods

The MCV1 coverage is calculated using the administrative method of dividing the total number of doses administered to children in the target age group by the census-estimated number of children in that age group, These national measles vaccination coverage data are reported annually to the WHO and UNICEF using the joint reporting form (JRF). WHO and UNICEF generate annual national estimates of coverage for each country.

For this analysis, we used the national MCV1 coverage estimates generated by WHO and UNICEF^10^, and as published in the latest DHS Survey^7^. The WHO UNICEF coverage estimates are not available for the year 2016 at the time of this analysis. We also analysed the administrative and survey coverage data from measles mass vaccination campaigns conducted between 2011 and 2016.

Since 2006, Nigeria has established nationwide measles case-based surveillance, identifying and reporting suspected measles cases, and testing serum samples from these cases for serological confirmation using standard WHO protocols and case definitions. Specimens with negative IgM results for measles and systematically tested for rubella IgM^3^. The national case-based surveillance database is shared with the WHO on a weekly basis.

The sensitivity of the surveillance system is regularly reviewed at the national and subnational levels using performance indicators. The principal monitoring indicators are: the annualized non-measles febrile rash illness rate (target of 2 cases per 100,000 population) and the proportion of districts that have reported at least 1 suspected case of measles with a blood specimen over a period of 1 year (target of 80%). We looked at the surveillance performance using the principal monitoring indicators, and analysed the epidemiological trends and patterns of occurrence of measles in Nigeria using the measles cases based surveillance data from 2012 – 2016.

## Results

The national MCV1 coverage reported through by Nigeria to the WHO and UNICEF was 78% in 2012, 73% in 2013 and 2014, and 71% in 2015. According to the WHO UNICEF annual vaccine coverage estimates, MCV1 coverage in Nigeria was between 42% and 54% in the years 2012 – 2015. Nigeria conducted three nationwide SIAs in the course of the last 5 years, at 2 years intervals. The 2011 SIAs achieved an administrative coverage of 91%, while the SIAs in 2013 and 2015 a reported coverage of more than 100%. Post-campaign coverage survey following the SIAs in 2013 and 2015 had national level coverage of 74.5% and 84.5% respectively. (Table 1)

In the past 5 years, Nigeria has reported a total of 114,323 suspected measles cases through the case based surveillance system, of which 82,266 (72%) were confirmed as measles by laboratory, epidemiological linkage or clinical compatibility. The number of confirmed measles cases peaked in 2013 and declined sharply in 2014. However, there was an increase in confirmed measles cases in 2014 and 2015. At the same time, there were 1,245 lab confirmed rubella cases, most of which were reported in 2015 and 2016. (Table 2).

The average incidence of confirmed measles in Nigeria ranged from 25 per million in 2014 to 303 per million population in 2013. Disaggregating the incidence levels by the State level shows that in the Northern States have significantly higher incidence levels as compared to the Southern States. An increase in age-specific incidence is observed in the age groups below 5 years and 5-9 years between 2014 and 2016 in the Northern States. Looking at the average incidence rate per million population across 5 years by State, the median incidence rate was 108.2 and 27.1 per million for the Northern and Southern States respectively. (Table 3)

The proportion of confirmed measles cases in the age group less than 5 years ranges between 65% and 83% of the annual number of confirmed cases, while children in the age group 5 – 9 years made up 14 – 22% of the total. (Table 4). The age specific incidence of measles was more than 520 and 120 per million population respectively for the under five and 5 – 9 years age groups in the Northern States in 2016. (Table 5)

The median proportion of unvaccinated children by State, among the confirmed measles cases ranged between 71% and 85% in the Northern States between 2012 and 2016, while it was 32.5% – 56% in the Southern States. (Table 4)

## Discussion

The immunisation coverage targets for measles elimination of 95% at national level and in every district are quite high and the interruption of endemic transmission can only be achieved with high population immunity. The WHO UNICEF coverage estimates for Nigeria indicate routine measles first dose vaccination coverage of less than 55% during 2012-2016, with a significant subnational level disparity as shown in the DHS 2013 data.

The post-campaign survey coverage of 75% and 84% following the SIAs in 2013 and 2015 respectively, also indicate gaps in SIAs quality while the SIAs coverage target is 95%, and are less than the post-SIAs survey coverage from many countries in the African Region^6^. With the large size of the under-five population targeted in nationwide SIAs in Nigeria, this coverage gap translates into hundreds of thousands of under-five children not being reached through SIAs.

According to the surveillance data, the high proportion of unvaccinated children among the confirmed measles cases indicating that gaps in service delivery (routine immunisation and SIAs) remains a major programmatic reason for the occurrence of high number of measles cases.

A previous report documented that Nigeria experienced a significant decline in measles incidence following the initial measles catch-up SIAs in 2005/ 2006, but later experienced resurgence in 2008^11^. The data for 2012 – 2016 shows a relatively high incidence throughout, with a marked spike in 2013, especially in the Northern States.

In 2016, Nigeria reported more than 11,800 confirmed cases. This accounts for nearly 40% of the 28,279 confirmed measles cases reported from the African Region in 2016^11^.

The incidence of measles in the Northern States continues to be quite high as compared to the Southern States. This is a reflection of the significant disparity in the coverage levels across the northern and southern zones of Nigeria. In addition, the immunity gaps created by low routine immunisation coverage could not be closed through SIAs as a result of suboptimal SIAs coverage. These gaps contribute to the epidemiological shift of measles towards older age groups. The proportion of confirmed measles cases reported in the age group above 5 years has been 17 – 35% across these years, with 19% of all cases occurring in children aged 5 – 9 years of age in 2016. This epidemiological picture requires detailed analysis and further action in the form of wide age range SIAs in order for the country to attain a reduction in measles incidence levels.

Despite the fact that the target for district reporting has been met every year since 2012, the surveillance performance has not been up to the Regional standards, with a non-measles febrile rash illness rate of less than 2 per 100,000 in 2014 and 2016. Countries in the African Region implement measles case-based surveillance integrated with AFP surveillance. The presence of a strong polio infrastructure and quite high AFP surveillance performance at subnational levels in Nigeria is an excellent opportunity to strengthen the levels of integration in case detection and reporting, technical support and supervision, as well as the monitoring of measles surveillance implementation in the country^12^.

As of 2017, Nigeria has not yet introduced rubella vaccine. The analysis of surveillance data has indicated that an increasing number of lab confirmed rubella cases are being detected in the country, with 465 confirmed cases in 2016. Further analysis of the rubella epidemiology and review of data related to the occurrence of congenital rubella syndrome will be necessary in order to provide the evidence for rubella vaccine introduction in the longer term. However, the country should establish sentinel surveillance for congenital rubella syndrome (CRS) soon in order to determine a baseline for the incidence of CRS, and to monitor the impact of rubella vaccine introduction at a later date.

In order to accelerate its progress towards the measles elimination targets, Nigeria should utilise all opportunities to prioritise and address the core and supportive elements of the immunisation system (including immunisation program financing, human resources, cold chain and vaccine management, etc) in order to scale up routine immunisation coverage equitably, especially in the Northern half of the country where the coverage is significantly low; introduce a second dose of measles vaccine in the routine schedule and achieve high coverage; tailor the conduct of measles SIAs in such a way as to boost the immunity levels of target populations, in order to sharply reduce measles incidence in age groups heavily affected by the disease; effectively mobilise resources and use available tools to improve on the quality of planning and coverage outcome of SIAs. In addition, the country should utilise the presence of a strong polio infrastructure to build solid systems for the surveillance for all vaccine preventable diseases.

This study has a few limitations. Administrative vaccination coverage for routine services and SIAs may not reflect the reality, since children beyond the target age group may be included in the numerator figures, and since the denominator data is often outdated or inaccurate. Not all suspected measles cases get notified and reported through the surveillance system. The serological laboratories in Nigeria have not been able to test all specimens because of periods of stock out of reagents in 2015 and 2016, leading to more reliance on epidemiological linkage to confirm measles cases.

## Figures and Tables

**Table 1:** MCV1 and SIAs coverage in Nigeria. 2012 – 2015.

	2012	2013	2014	2015
MCV1 coverage (WHO UNICEF coverage estimates)	42%	47%	51%	54%
Officially reported MCV1 coverage	78%	73%	73%	71%
Measles follow up SIAs – # of children reached		30,579,666		43,134,811
Measles follow up SIAs – administrative coverage results		103.1%		110.1%
Measles follow up SIAs - Post SIAs coverage survey results		74.5%		84.5%

**Table 2:** Measles case based surveillance performance and reported cases. Nigeria. 2012 - 2016.

	2012	2013	2014	2015	2016
Non-measles Febrile Rash Illness Rate per 100,00 population	2.8	2.2	1.4	2.1	1.8
Proportion of districts reporting at least 1 case with blood specimen per year	86%	92%	81%	90%	93%
Suspected measles cases	10,959	59,025	9,343	15,031	19,965
Confirmed measles cases	5,189	52,900	4,483	7,857	11,837
Confirmed rubella cases	228	70	89	393	465
Incidence of confirmed measles per million population	31	303.2	24.9	42.3	61.7

**Table 3:** Annual incidence of confirmed measles reported through the case based surveillance system in Northern and Southern Nigeria. 2012 - 2016.

		2012	2013	2014	2015	2016
Annual incidence of confirmed measles per million population	Northern States	61.6	540.6	46.3	100.4	112.2
	Southern States	10.4	60	28.5	27.9	2.8

**Table 4:** Age group and vaccination status of confirmed measles cases reported through the case based surveillance system. Nigeria. 2012 - 2016.

	2012	2013	2014	2015	2016
Age group of confirmed measles cases					
< 5 years	2,238 (72%)	43,223 (83%)	2,835 (65%)	5,961 (78%)	8,674 (75%)
5 - 9 years	664 (21%)	7,373 (14%)	975 (23%)	1,277 (17%)	2,233 (19%)
10 - 14 years	122 (4%)	1,265 (2%)	301 (7%)	219 (3%)	385 (3%)
15 years plus	80 (3%)	331 (1%)	232 (5%)	145 (2%)	334 (3%)
Vaccination status of confirmed measles cases					
unknown	91 (1.4%)	2483 (4.5%)	670 (9.8%)	1022 (8.2%)	1572 (12.2%)
unvaccinated	4984 (77.9%)	45339 (81.7%)	3671 (53.5%)	7937 (63.8%)	7889 (61.1%)
1 or more doses	1322 (20.7%)	7695 (13.9%)	2523 (36.8%)	3479 (28.0%)	3447 (26.7%)

**Table 5:** Age specific incidence of confirmed measles per million population by age group for different parts of the country. 2016. Nigeria.

	<5 Yrs	5 - 9 Yrs	10 - 14 yrs	15 - 19 yrs	20 yrs and above
Northern States	526.8	151.9	30.8	13.9	3.4
Southern States	10.6	3.8	2.1	1.3	0.5
